# Spatio-Temporal Patterns of the 2019-nCoV Epidemic at the County Level in Hubei Province, China

**DOI:** 10.3390/ijerph17072563

**Published:** 2020-04-08

**Authors:** Wentao Yang, Min Deng, Chaokui Li, Jincai Huang

**Affiliations:** 1 National-local Joint Engineering Laboratory of Geospatial Information Technology, Hunan University of Science and Technology, Xiangtan 411100, China; yangwentao8868@126.com (W.Y.); chkl_hn@163.com (C.L.); 2 Hunan Provincial Key Laboratory of Geo-information Engineering in Surveying, Mapping and Remote Sensing, Hunan University of Science and Technology, Xiangtan 411100, China; 3 School of Geosciences and Info-physics, Central South University, Changsha 410083, China; huangjincaicsu@csu.edu.cn; 4 Shenzhen Key Laboratory of Spatial Smart Sensing and Service, Shenzhen University, Shenzhen 518060, China

**Keywords:** 2019 novel coronavirus, geographic information science, abrupt change, spatial cluster, spatial outlier, daily new confirmed cases, incidence rates

## Abstract

Understanding the spatio-temporal characteristics or patterns of the 2019 novel coronavirus (2019-nCoV) epidemic is critical in effectively preventing and controlling this epidemic. However, no research analyzed the spatial dependency and temporal dynamics of 2019-nCoV. Consequently, this research aims to detect the spatio-temporal patterns of the 2019-nCoV epidemic using spatio-temporal analysis methods at the county level in Hubei province. The Mann–Kendall and Pettitt methods were used to identify the temporal trends and abrupt changes in the time series of daily new confirmed cases, respectively. The local Moran’s I index was applied to uncover the spatial patterns of the incidence rate, including spatial clusters and outliers. On the basis of the data from January 26 to February 11, 2020, we found that there were 11 areas with different types of temporal patterns of daily new confirmed cases. The pattern characterized by an increasing trend and abrupt change is mainly attributed to the improvement in the ability to diagnose the disease. Spatial clusters with high incidence rates during the period were concentrated in Wuhan Metropolitan Area due to the high intensity of spatial interaction of the population. Therefore, enhancing the ability to diagnose the disease and controlling the movement of the population can be confirmed as effective measures to prevent and control the regional outbreak of the epidemic.

## 1. Introduction

As a result of the outbreak of pneumonia caused by the 2019 novel coronavirus (2019-nCoV), Wuhan, the capital of Hubei province in China, became a focus of global attention. After the first case of coronavirus was reported in Wuhan in December 2019, which was not the source of the virus, the coronavirus spread to domestic and foreign areas, resulting in a significant threat to the world. The 2019-nCoV situation reports, published by the World Health Organization (WHO) on 25 February 2020, show that 77,780 cases had been confirmed in China. Outside of China, the number of confirmed cases was 2459, and 2700 infected patients had died [1]. Consequently, the WHO has classified the 2019-nCoV epidemic as a public health emergency of international concern. China and other affected countries have adopted a series of response measures to prevent and control this extraordinary public safety event [2]. 

Indeed, to effectively prevent and control this epidemic, one of the most critical tasks is to clearly and comprehensively understand the epidemic from different perspectives. Currently, a large body of research has been accomplished, especially in the field of medicine. Mainly, these investigations focus on the genomic characterization of the novel coronavirus [3,4], the clinical characterization of the infected patients [5,6], and the medical diagnosis and treatment of the infected cases [7,8]. The medical research immensely contributes to the recognition of the virus’ characteristics and to obtaining the cure. However, no study has discussed the spatio-temporal patterns of the 2019-nCoV epidemic. Mainly, the spatio-temporal patterns describe the epidemical characteristics, including spatio-temporal distribution (regular, clustered, or random), spatio-temporal association, and spatio-temporal evolution [9]. This is beneficial for enhancing the understanding of this epidemic in the spatio-temporal dimension and providing reliable information for decision-making. For example, Liu et al. [10] and Dong et al. [11] analyzed the spatial and temporal characteristics of human infection of avian influenza A(H7N9) in mainland China in 2013. Their results showed that there existed spatially clustered characteristics. Meanwhile, the research by Qiu et al. [12] identified additional risk factors. To understand the dynamic spread of porcine epidemic diarrhea in 2013 in the US, global and local analysis methods uncovered the spatio-temporal patterns of this epidemic [13]. The spatio-temporal distribution and diffusion pattern of the dengue outbreak in Swat, Pakistan, in 2013, was also discovered based on the spatio-temporal analysis method, and the implications were assessed and recommendations were made [14]. Spatio-temporal patterns (i.e., trends and clusters) of influenza in 2005–2018, in China, were detected by spatio-temporal analysis where high-risk areas and the corresponding reasons were identified [15].

As a result, this research aims to detect spatio-temporal patterns of the 2019-nCoV epidemic by using spatio-temporal analysis methods, namely, nonparametric statistical tests implemented by Python and spatial autocorrelation indexes implemented in ArcGIS 10.2. Additionally, considering that apart from Wuhan, where the epidemic first arose, other areas inside the Hubei were also severely affected, we focus on spatio-temporal patterns at the county level in Hubei province. The structure of the article is organized as follows: Section 2 describes the study area, datasets, and methods; Section 3 presents and discusses the results of the identified spatio-temporal patterns and the possible reasons; then, Section 4 provides a summary of our work and details future work. 

## 2. Materials and Methods 

### 2.1. Study Area and Dataset

The study area, i.e., Hubei province, is located in the middle reaches of the Yangtze River and the central part of China. It ranges from 29°01′N to 33°06′N latitude and 108°21′W to 116˚08′W longitude, with a total area of about 185,900 km^2^. Hubei is characterized by a subtropical climate and has four distinct seasons with cold winters and scorching summers. The annual average temperate is about 15 °C and the coldest and hottest months are January (i.e., averaging 1–5 °C) and July (i.e., averaging 27–30 °C), respectively. The annual precipitation is between 800–1600 mm and the wettest month in Hubei is summer with 300–700 mm of rainfall.

**Figure 1:**
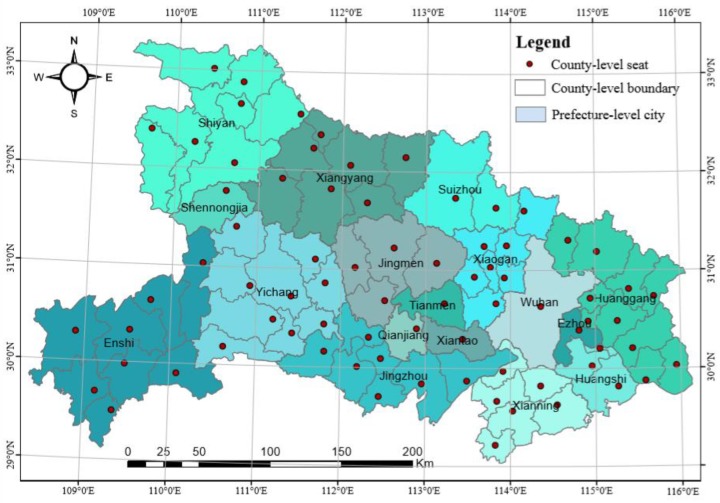
The map of the study area in Hubei province, China.

The dataset for this study is composed of the epidemic data and the auxiliary data. The epidemic data mainly includes the number of daily confirmed cases and a total number of regular cumulative confirmed cases (the statistical period is from 0:00 to 24:00). These cases were directly collected by the local Health Commission and reported on the local government website of each city. The auxiliary data include the disease control data also derived from the local government website and the total population collected from China population statistic yearbook (2017). A relatively complete epidemic dataset was found in 75 county-level cites. The spatial distribution of these county-level cities and their corresponding prefecture-level cites are shown in Figure 1. 

We only considered the dataset from January 26 to February 11 for the current study. This is because from February 12, the criteria for confirmed cases, in Hubei, was changed by adding clinically diagnosed cases that show signs of pneumonia in the lungs of patients based on a computerized tomographic scan. The 13,332 clinically diagnosed cases on February 12 were added to the total number of daily new confirmed cases, which was far higher than those on February 11 (1638). The change in criteria would result in variations in spatio-temporal patterns for most areas. 

### 2.2. Methods

**Figure 2:**
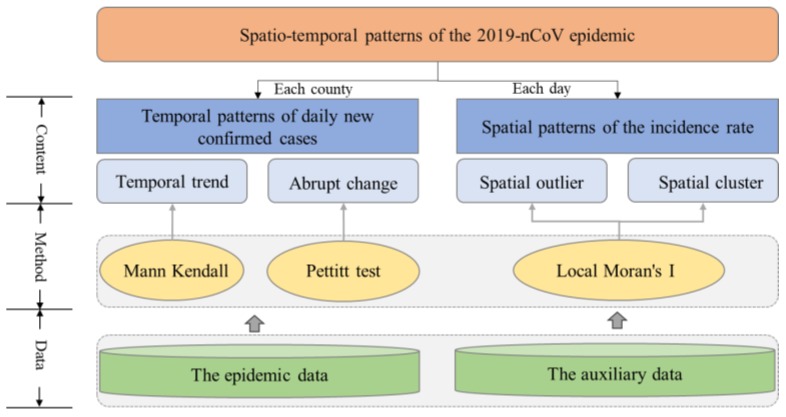
The main analytical framework used in this study.

As shown in Figure 2, this research aims to discover the spatio-temporal patterns of the 2019-nCoV epidemic from two aspects, i.e., temporal and spatial patterns. The temporal pattern, i.e., of daily new confirmed cases per county, is defined based on the characteristics of the temporal trend and abrupt change. In contrast, spatial patterns, i.e., of the incidence rate per county per day, include spatial outliers and clusters. Two types of spatio-temporal analysis methods, namely, nonparametric statistical methods and geographical analysis methods, were used to achieve the above aim. Specifically, Mann–Kendall and Pettitt tests were used to test the temporal trend and abrupt changes in a time series of daily new confirmed cases, respectively. The local Moran’s I index was applied to identify spatial outliers and clusters. A brief introduction to the above methods is given in the following subsection.

#### 2.2.1. Identifying Temporal Patterns Using the Nonparametric Test

As mentioned earlier, the temporal patterns of daily new confirmed cases were determined by two characteristics: temporal trend and abrupt change. A temporal trend is a continued increase or decrease in the time series of the number of daily new confirmed cases that can describe the epidemical risk for a given period. Generally, the trend changes smoothly or regularly and can be seen from the commonly used epidemic models [16]. However, owing to the interference of specific events (such as large-scale vaccination and isolating suspected cases), an abrupt change in the time series of daily new confirmed cases may occur. Therefore, based on the level of significance of these trends (i.e., increasing or decreasing trends) and significant abrupt change, six types of temporal patterns can be defined (see Table 1). The ITAC pattern has an Increasing Trend and an Abrupt Change, while the ITNO pattern has an Increasing Trend only, and the DTAC pattern has a Decreasing Trend and an Abrupt Change. Moreover, the DTNO pattern has a Decreasing Trend only, while the NOAC pattern has Abrupt Change only, and the NONO pattern has none of these two characteristics.

The Mann–Kendall (M–K) test, a nonparametric method, is widely applied to identify a monotonous trend in a time series [17,18,19]. It does not require samples to conform to a specific statistical distribution and has a good performance despite several outliers in the series. An M–K *Z* statistic that follows a standard normal distribution was used to test the trend. Assuming that (*x*_1_, *x*_2_, …, *x_n_*) construct a time series, where *x_j_* represents the sample collected at time *j*, the M–K statistic *S* is defined as

(1) $$S=\overset{n}{\underset{i=2}{\sum }}\overset{i-1}{\underset{j=1}{\sum }}Sign\left(x_{j}-x_{i}\right)$$

where

(2) $$Sign=\{\begin{array}{c} 1\;\;\;x_{j}-x_{i}>0\\ 0\;\;\;x_{j}-x_{i}=0\\ -1\;\;x_{j}-x_{i}<0 \end{array}$$

Provided there are not many tied values within the time series with a length of more than 10, the statistic *S* approximately conforms to the normal distribution. Further, a normal *Z* statistic test by standardizing the statistic *S* can be applied for datasets with no less than 10 values. The normal *Z* statistic is as follows:

(3) $$Z=\{\begin{array}{c} \frac{S-1}{\sqrt{Var\left(S\right)}}\;\;S>0\\ 0\;\;\;S=0\\ \frac{S+1}{\sqrt{Var\left(S\right)}}\;\;S<0 \end{array}$$

where *Var*(*S*) is the variance of *S*, expressed as

(4) $$Var\left(S\right)=\left[n\left(n-1\right)\left(2n+5\right)-\sum_{k}^{m}t_{k}\left(t_{k}-1\right)\left(2t_{k}+5\right)\right]/18$$

where *n* is the number of samples or the length of the time series; *m* is the number of tied groups (a tied group denotes a set of samples that have the same value); and $t_{k}$ is the number of samples in the kth tied group. For instance, concerning the time series (2, nondetect, 2, 2, nondetect, 3, 5, 4, 4, 5), it can be seen that *n* = 10, *m* = 4, *t*_1_ = 2 for the nondetects, and *t*_2_ = 3 for the tied value 2. A positive value of *Z* statistic indicates an increasing trend. On the contrary, a negative value corresponds to a decreasing trend. Otherwise, there is no significant monotonous trend. The null hypothesis, H_0_, for the test is that there is no trend in the series, while the alternative hypothesis is that there is a non-null (i.e., negative or positive) trend. When the absolute value of the *Z* statistic exceeds 1.64, the trend is significant at a confidence level of 95%, which was selected for this study.

The Pettit test, i.e., a rank-based and distribution-free test, is commonly used to identify the date of a change point in a time series [20,21,22]. For the sequence of random variable (*x*_1_, *x*_2_, …, *x_n_*) with a change point at τ, the common distribution function for *x_t_* (*t* = 1, 2, …, *τ*) is different from that for *x_t_* (*t* = *τ* + 1, *τ* + 2, …, *n*). The null hypothesis, H_0_, for this test is that no change or *τ* = *n* is tested against the alternative hypothesis that change or 1 ≤ *τ* < *n* by using the statistic *K_n_*, whose form can be described as

*Kn* = *Max*|*U_t_*,*_n_*|, 1 ≤ *τ* ≤ *n* (5)

where

(6) $$U_{t-n}=\overset{t}{\underset{i=1}{\sum }}\overset{n}{\underset{j=t+1}{\sum }}Sign\left(x_{i}-x_{j}\right)$$

The significance level associated with *K_n_* can be expressed as

(7) $$P≅2exp\left(\frac{-6k^{2}}{n^{3}+n^{2}}\right)$$

where *P* represents the probability of the presence of an abrupt change point in the time series.

#### 2.2.2. Discovering Spatial Patterns by Local Moran’s I Index

Moran’s I index is a measure of spatial autocorrelation or dependency that can be used to explore the spatial structure of infectious diseases [23,24,25]. Autocorrelation always shows significant local variations because of spatial heterogeneity in spatial or epidemical data. Consequently, local Moran’s I index was used to explore the local spatial autocorrelation or dependency of the spatial data [26]. 

Assuming that (*x*_1_, *x*_2_, …, *x_n_*) represents data observed at different spatial locations, the form of the local Moran’s I index at spatial location *i* can be expressed as

(8) $$I_{i}=\frac{x_{i}-\bar{x}}{S_{i}^{2}}\overset{n}{\underset{j=1,j\neq i}{\sum }}w_{ij}\left(x_{j}-\bar{x}\right)$$

where $\bar{x}$ is the mean value of all the spatial data; $w_{ij}$ represents spatial weight between the data at spatial location *i* and *j*; and $S_{i}^{2}$ can be calculated as

(9) $$S_{i}^{2}=\frac{\sum_{j=1,j\neq i}^{n}{\left(x_{j}-\bar{x}\right)}^{2}}{n-1}-{\bar{x}}^{2}$$

Further, a normal $Z_{i}$ statistic test derived from standardizing the statistic $I_{i}$ can be applied to perform the hypothesis testing. The null hypothesis, H_0_, shows that data is independent at spatial location *i*, while the alternative hypothesis, H_1_, shows that autocorrelation structure exists at this location. The $Z_{i}$ statistic can be expressed as

(10) $$Z_{i}=\frac{I_{i}-E\left(I_{i}\right)}{\sqrt{V\left(I_{i}\right)}}$$

where

(11) $$E_{i}=-\frac{\sum_{j=1,j\neq i}^{n}w_{ij}}{n-1}$$

And

(12) $$V\left(I_{i}\right)=E\left(I_{i}^{2}\right)-E{\left(I_{i}\right)}^{2}$$

On the basis of the $Z_{i}$ statistic value, four types of spatial patterns were defined, namely, the High–High (HH) cluster, High–Low (HL) outlier, Low–Low (LL) cluster, and Low–High (LH) outlier. When the $Z_{i}$ statistic is larger than 1.64 (i.e., with a confidence level of 0.05), a unit with a large value of *x_i_* corresponds to an HH cluster, and a unit with a small value of *x_i_* is defined as an LL cluster. Similarly, when the $Z_{i}$ statistic is smaller to −1.64, a unit with a large value of *x_i_* is identified as an HL outlier, and a unit with a small value of *x_i_* is regarded as an LH outlier.

These four types of spatial patterns can reveal the spatial structure of the incidence rate of the epidemic in the study area. Specifically, an HH (LL) cluster indicates several adjacent areas with a relatively high (low) value of incidence rate of the epidemic, which can reveal a high (low) risk of epidemic in these areas. An HL (LH) outlier means a high (low) value surrounded primarily by low (high) values of incidence rate, which may be caused by a unique mechanism [27]. On this basis, a dynamic spatial pattern can be identified for the incidence rate of all the cities at different times.

## 3. Results and Discussion

### 3.1. Identifying the Temporal Patterns of Daily New Confirmed Cases

**Figure 3:**
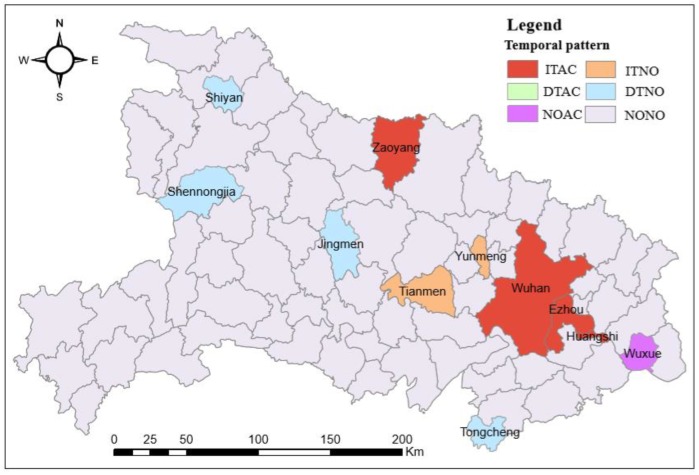
Temporal patterns of the number of daily new confirmed cases.

According to the results (Table 1), temporal patterns of daily new confirmed cases were obtained in this study area (see Figure 3). Eleven areas showed significant temporal patterns during the study period, and four areas (i.e., Wuhan, Ezhou, Huanggang, and Zaoyang) with ITAC patterns, two areas (i.e., Yunmeng and Tianmen) with ITNO patterns, one area (i.e., Wuxue) with a DTAC pattern, and four areas (i.e., Shiyan, Shennongjia, Jingmen, and Tongcheng) with DTNO patterns.

Almost all the areas (except Zaoyang) that showed increasing trends (including ITAC and ITNO patterns) are distributed near Wuhan. After confirming that the 2019-nCoV epidemic could spread among people, Wuhan was sealed in an emergency to control the transmission of infections from January 23, 2020. Wuhan, the capital of Hubei province, is situated in east-central Hubei and constitutes over a fifth of the province’s population. It is recognized as the political, economic, financial, cultural, educational, and transportation center of central China. Because Wuhan is also one of the largest integrated transportation and communication hubs in China, there is a large amount of population interaction and mobility between Wuhan and other areas, both inside and outside of the province. The big data map of migration published by Baidu (https://voice.baidu.com/act/newpneumonia/newpneumonia/?from=osari_pc_1) shows that the destinations of most of the population departing from Wuhan before January 23 are the cities or counties inside Hubei province, especially its surrounding areas. Because of the incubation period of 0 to 14 days and the mean period of 6.4 days [28], quite a few virus carriers did not receive treatment in isolation and the phenomenon of human-to-human transmission was not effectively controlled during a specific period after January 23. Most of the surrounding areas had to face the difficulty of an increasing trend of daily new confirmed cases. Compared to areas showing increasing trends, the areas with decreasing trends (i.e., DTAC and DTNO) were randomly distributed. 

**Figure 4:**
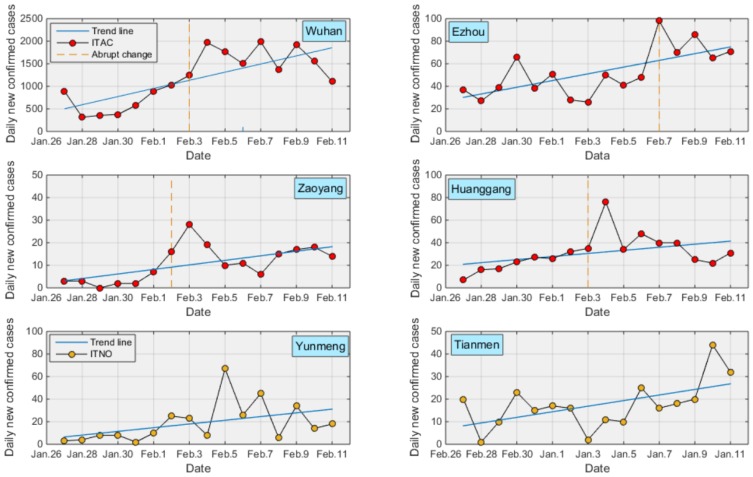
The change curve of these areas with the ITAC or ITNO patterns.

The change in the curve in the areas with the ITAC or ITNO pattern is shown in Figure 4. The abrupt change date of daily new confirmed cases in Wuhan was February 3. Before this day, the number of daily new confirmed cases was approximately 400 to 1,000, and after this day, the numbers fluctuate around 1750. One possible reason is that the number of infected cases was still increasing. However, a more important reason is that on February 2, as a result of the insufficient diagnostic resources, there were lots of suspected cases that had not yet been confirmed. The local government emphasized that they must enhance the ability to examine the infection by several measures, such as providing full payment to third-party examiners, simplification of the examination process, and strengthening the quality of sample allocation and inspection. The abrupt change in Ezhou, on February 7, can be attributed to similar factors to those mentioned above, namely, the improvement of the ability of the medical service. Ezhou was one of the most severely affected areas, and the lack of medical workers greatly affected diagnosis and treatment. On account of this, on February 4, a proposal published by the local disease command invited the retired, resigned, and independent medical workers to return to work. Meanwhile, a medical team from Peking University International Hospital arrived in Ezhou to participate in disease control on February 7.

The abrupt change date of the other two areas (i.e., Zaoyang and Huangshi) with the ITAC pattern is approximately located on the date with the maximum number of daily new confirmed cases. The disease command at Zaoyang reported that from February 2, all suspected cases would be examined within a few days so that the number of these cases would be decreased to 0. For Huangshi, the number of institutions authorized to conduct nucleic acid tests increased from 4 to 6 from February 3. Although Yunmeng and Tianmen with the ITNO pattern did not show any significant abrupt changes, an increasing short-term trend appeared at Yunmeng after February 5. 

For these areas with DTAC or DTNO, decreasing trends imply that the epidemic was almost under control. All the remaining areas had NONO, which indicates that the daily new confirmed cases in most of these areas generally fluctuated in a particular range and thus did not have significant abrupt change points. Moreover, it can be deduced that a short-time trend, in this study, cannot be identified because the M–K test can only investigate a monotonous global trend over the entire study period. Thus, this was the limitation of the study. For instance, although the daily new confirmed cases of Yunmeng exhibit an increasing trend (see Figure 4) over the whole period, there was also a decreasing trend after February 5.

### 3.2. Discovering the Spatial Patterns of the Incidence Rate 

**Figure 5:**
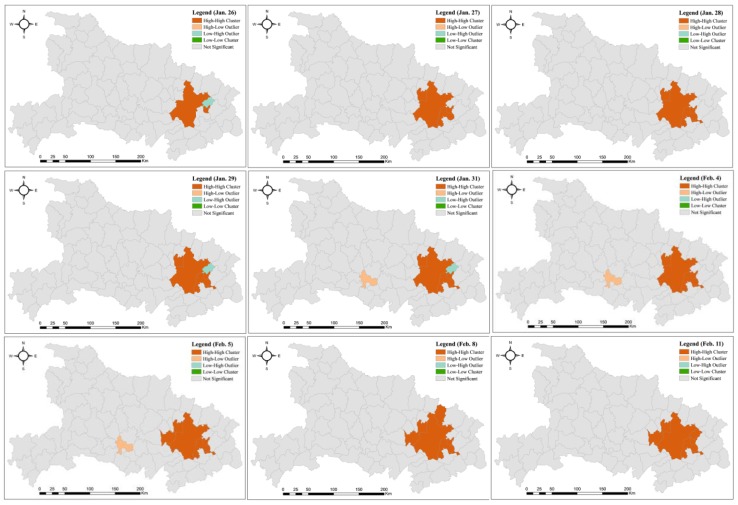
The dynamic process of the spatial patterns of the incidence rate.

The incidence rate of all areas at the county level was calculated based on the population data and the total number of infections. To obtain the evolution process, local Moran’s I indices for each day during the period (January 26 to February 11) were calculated using ArcGIS 10.2. The spatial weight, in Equation (8), was computed based on the edge contiguity of these areas, i.e., *w_ij_* is equal to 1 if there is a shared edge between two areas *i* and *j*, and otherwise, *w_ij_* is equal to 0. Further, on the basis of the statistical value at the confidence level of 0.05, spatial patterns of the incidence rate were identified (Figure 5). If the pattern on a given day is the same as on the previous day, then the pattern on the current day is not shown. Overall, from Figure 5, it can be found that this study discovered only three from the dataset, namely, HH clusters, HL outliers, and LH outliers. The proportion of HH clusters among all identified patterns was the highest, i.e., mainly around Wuhan, followed by HL clusters. 

Notably, on January 26, the areas, including Wuhan and Huanggang, where the incidence rates were 0.071‰ and 0.361‰, respectively, were first detected as HH clusters. Although the total number of infections in Wuhan was more significant than in Huanggang, the population of Huanggang is much smaller than that of Wuhan. Thus, this resulted in a higher incidence rate. Subsequently, with the increase of confirmed cases in each area, the incidence rate also increased. The areas near Wuhan and Huanggang, such as Xiaogan (January 27), Ezhou (January 27), Huangshi (January 28), Hanchuan (February 5), Hongan (February 8), and Tuanfeng (February 11), were recognized successively as an element of the HH cluster. As mentioned above, all these areas near Wuhan received most of the population departing from Wuhan, which led to high incidence rates.

All the LH outliers appeared in Tuanfeng on January 26, and from January 29 to February 3. Tuanfeng, with a low number of infections (i.e., less than 5), shows an outlier characteristic because it was near the HH cluster before February 3. However, from February 3 to February 11, the total number of infections increased from 50 to 117; the high incidence rate resulted in the HH cluster due to proximity. Similarly, Jinzhou was identified as a unique area with the HL outlier from January 31 to February 7. During this period, the number of confirmed cases increased from 149 to 312, which is significantly higher than the surrounding area, thus leading to an HL outlier.

## 4. Conclusions 

This research applied spatio-temporal analysis methods to detect spatio-temporal patterns of the 2019-nCoV epidemic at the county level in Hubei province. We mainly focused on temporal patterns of daily new confirmed cases and spatial patterns of the incidence rate from January 26 to February 11, 2020.

Regarding temporal patterns, most areas during the study period did not show significant characteristics of trend or abrupt change. Nevertheless, 11 areas with different types of patterns were detected, and these areas with increasing trend characteristics, included ITAC and ITNO patterns, are characterized by spatial aggregation. Notably, we found that the main reason for the abrupt change was the improvement of the nucleic acid examination capabilities when the number of suspected cases exceeded the detection capacity. Therefore, enhancing the capability to diagnose infections helps control the epidemic; otherwise, isolating the suspected patients is also an effective control measure.

For spatial patterns, spatial clusters with high incidence rates were detected during the whole study period. These areas are concentrated around Wuhan and also belong to the Wuhan Metropolitan Area or Greater Wuhan. As the political, economic, financial, cultural, educational, and transportation center of Greater Wuhan, a large population flow resulted in the high risk in Greater Wuhan. Spatial outliers were also discovered in two areas, i.e., Jingzhou and Tuanfeng, with a high–low outlier, and a low–high outlier, respectively. Because of spatio-temporal variation of daily new confirmed cases, spatial outliers did not appear during the whole period. Therefore, timely control of regional population flows would be beneficial to prevent a regional outbreak of disease.

However, the limited reliability of the data (for example, some infections require nucleic acid testing a minimum of two times (on two days), which results in biased data) may affect the analysis result. As a result of the limitation of the methods, we only discussed a few characteristics or patterns. Other characteristics that can be analyzed in depth include a short-term trend or turning points (which cannot be identified using the nonparametric tests used in this study), as well as the future risk in different areas. Additionally, descriptive and nonparametric methods can only detect spatio-temporal patterns. More reliable and quantitative methods need further exploration. For example, spatial regression models, such as geographically weighted regression [29] and spatial dynamic panel data models [30], can be used, which may also be a critical aspect in future research.

## Figures and Tables

**Table 1:** Different types of temporal patterns defined in this study.

Abrupt Change	Temporal Trend		
	Increasing Trend	Decreasing Trend	*Not Significant*
	*Significant*	*Significant*	
*Significant*	ITAC	DTAC	NOAC
*Not Significant*	ITNO	DTNO	NONO
